# A rapid visual detection method for indel polymorphisms in the bovine PRNP gene based on a duplex MIRA-LFD assay

**DOI:** 10.3389/fvets.2026.1798131

**Published:** 2026-05-01

**Authors:** Ye Xu, Siyu Yang, Siling Ding, Ting Xu, Like Zhu, Weiming Xiao, Jian Ge, Feng Guan

**Affiliations:** 1 College of Life Sciences, China Jiliang University, Hangzhou, China; 2 Key Laboratory of Dairy Cow Genetic Improvement and Milk Quality Research of Zhejiang Province, Wenzhou, China

**Keywords:** bovine, genetic polymorphism, MIRA-LFD, PRNP, visual detection

## Abstract

Insertion/deletion (indel) polymorphisms in the bovine *PRNP* gene promoter region (23 bp) and intron 1 (12 bp) influence gene expression and susceptibility to bovine spongiform encephalopathy (BSE). Conventional detection methods based on DNA sequencing are cumbersome and time-consuming. This study developed a visual detection method for these two polymorphic loci using multienzyme isothermal rapid amplification (MIRA) combined with lateral flow dipstick (LFD) technology, enabling efficient, low-cost, and rapid analysis. We designed specific MIRA primers and LFD probes for the bovine *PRNP* gene and optimized the reaction system. This allows the 12 bp and 23 bp indel polymorphisms to be detected visually based on the presence or absence of colored bands on test strips. By employing specific probes for each locus on a single duplex strip, the assay facilitates the simultaneous visual detection and haplotyping of both polymorphic loci. Tests on 62 randomly selected commercial cattle-derived products showed that the genotyping results for both loci were completely consistent with sequencing results. The established method is simple, convenient, highly specific, and easy to operate. It has minimal requirements for laboratory equipment, providing a practical technical platform for *PRNP*-assisted breeding in cattle and for the genotyping analysis of cattle products.

## 1 Introduction

Bovine spongiform encephalopathy (BSE), commonly known as “mad cow disease,” is a fatal neurodegenerative disorder caused by conformational changes in the prion protein (PrP) (1, 2). First identified in the United Kingdom in 1986, BSE later spread rapidly worldwide, causing severe losses to the livestock industry and triggering a significant public health crisis (3–7). The pathogenic prion protein is highly resistant to conventional disinfection and high-temperature treatments. Transmission can also occur through multiple routes, such as milk and meat products (2, 8–11), and even through environmental prion contamination (12, 13). These factors make pathogen eradication extremely difficult (12, 14–16) and pose ongoing risks to human health.

The bovine prion protein is encoded by the prion protein gene (*PRNP*), whose polymorphisms strongly influence BSE susceptibility and inheritance (17–21). Since the gene's full-length sequencing in 2001, studies have confirmed that the 23-bp indel in the promoter region and the 12-bp indel in the intronic region are significantly associated with resistance to BSE(7, 19, 22, 23). These loci serve as important molecular markers for breeding resistant individuals (17, 21, 24–27). Recent studies also indicate that disease resistance can be enhanced through genetic editing of the *PRNP* gene (28). Therefore, detecting *PRNP* gene polymorphisms to evaluate individual resistance to BSE provides a basis for disease-resistant breeding and can help guide control strategies. This approach is essential for protecting the livestock industry and safeguarding public health.

Current genotyping methods for the two indel polymorphisms in the *PRNP* gene primarily include Sanger sequencing (21, 29–34), PCR-RFLP (35), TaqMan probe-based qPCR (36), next-generation sequencing (NGS) (37), and microarray genotyping (38). However, these approaches generally require long processing times, are costly, and often depend on expensive instrumentation, making them unsuitable for routine use in basic laboratories. There is therefore an urgent need for a simple, low-cost, rapid, and reliable detection method that can improve the efficiency of *PRNP* polymorphism identification while maintaining accuracy and enabling result visualization.

Multienzyme isothermal rapid amplification (MIRA) is a recently developed isothermal amplification technology based on recombinase polymerase amplification (RPA). Following optimization of its enzyme system, MIRA demonstrates higher amplification efficiency and sensitivity (39–42). The MIRA reaction rapidly amplifies target fragments at a constant temperature (37–42 °C) (43). MIRA-LFD is an integrated detection platform that couples MIRA with lateral flow dipsticks (LFD), allowing visual interpretation of amplification products (44–46) and significantly improving detection convenience. These advances make the technology particularly suitable for applications in primary healthcare diagnostics, import-export quarantine, and outbreak response, offering an innovative solution for point-of-care molecular diagnostics.

Using MIRA-LFD technology, this study designed and optimized specific primer-probe sets targeting the 23 bp and 12 bp indel loci in the bovine *PRNP* gene to establish genotyping assays for both sites. This work aims to further enhance detection efficiency, expand existing genotyping methodologies, and provide technical support for gene-assisted breeding and disease risk assessment in cattle.

## 2 Materials and methods

### 2.1 Sample sources and DNA extraction

A total of 230 genomic DNA samples were available from laboratory archives. These were isolated from blood samples of *Holstein cattle* (*n* = 200), *Yellow cattle* (*n* = 10), and *Wenzhou water buffalo* (*n* = 20). Furthermore, commercial specimens (including fresh milk, beef, milk powder, and cheese; *n* = 62) were purchased from retail markets, representing diverse production batches and dates. The blood samples of *Holstein cattle* and *Wenzhou water buffalo* were provided by the Key Laboratory of Dairy Cow Genetic Improvement and Milk Quality Research of Zhejiang Province, while those of *Yellow cattle* were from the Dairy Cattle Breeding Farm of Shandong Academy of Agricultural Sciences. Genomic DNA was extracted using a commercial Animal Tissue DNA Extraction Kit (Hangzhou Xinjing Biotechnology Co., China). All animal-related procedures were approved by the Animal Experiment Ethics Committee of China Jiliang University (Approval No. 2021-005).

Genomic DNA was extracted from these commercial specimens using the TakaRa MiniBEST Universal Genomic DNA Extraction Kit (Takara Bio, China) according to the manufacturer's instructions. Briefly, approximately 25 mg aliquots of each specimen underwent the manufacturer-specified pretreatment, followed by genomic DNA isolation as directed. The concentration and purity of the extracted DNA were quantified using a NanoDrop 2,000 spectrophotometer (Thermo Fisher Scientific). DNA integrity was assessed by 1.5% agarose gel electrophoresis with GelRed staining.

### 2.2 Sequencing analysis of two PRNP polymorphic loci

The genomic DNA samples used for Sanger sequencing validation were randomly selected from the 230 bovine and water buffalo samples described in Section 3.1 (including Holstein cattle, Yellow cattle, and Wenzhou water buffalo). The 12 bp and 23 bp indel polymorphisms were sequenced and validated using primer pairs previously reported by Imran et al. (47), with the bovine *PRNP* gene sequence (GenBank accession No. AJ298878.1) as the reference. The target polymorphic sequences were defined as 5'-GGGGGCCGCGGC-3'= (12 bp) and 5'-TCTCAGATGTCTTCCCAACAGCA-3' (23 bp). All primers were synthesized by Hangzhou Tsingke Biotechnology Co., Ltd. (China), and their sequences are listed in Table 1.

**Table 1:** Bovine PRNP gene PCR sequencing primers.

Polymorphic locus	Primer	Primer sequence (5' → 3')
12bp indel	12indel F	GTGCTCGTTGGTTTTTGC
	12indel R	TCCTACACACCACCCACATA
23bp indel	23indel F	AGCCAGGTAAGAAGCTCATC
	23indel R	CATGAATTGTGTAGGCCAAA

PCR amplification for the 12 bp and 23 bp indel fragments was conducted separately using two independent primer sets under identical reaction conditions. Each 20 μl reaction followed the same thermal profile: initial denaturation at 95 °C for 5 min; 35 cycles of denaturation at 94 °C for 30 s, annealing at 60 °C for 30 s, and extension at 72 °C for 45 s; followed by a final extension at 72 °C for 10 min. The reaction was then held at 4 °C. Amplification products were separated by electrophoresis on 1.5% agarose gels. Bands corresponding to the expected sizes were excised, purified, and sent to Hangzhou Tsingke Biotechnology Co., Ltd. (Hangzhou, China) for bidirectional Sanger sequencing. Sequence editing and alignment were performed using EditSeq and MegAlign software, followed by comparison against the NCBI database via BLAST (https://blast.ncbi.nlm.nih.gov/Blast.cgi).

### 2.3 Design of MIRA primers and probes

**Figure 1:**
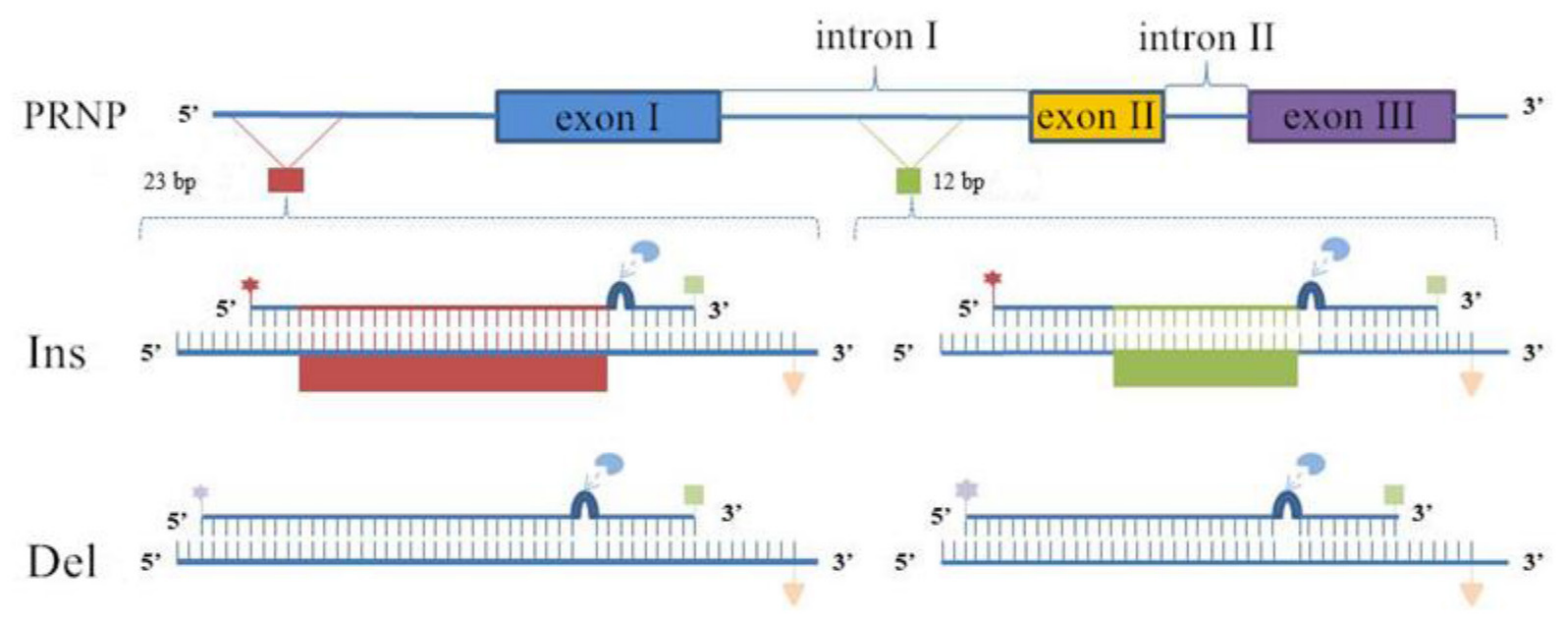
Design of MIRA Primers and Probes for the 12 bp and 23 bp Loci of the PRNP Gene. The reverse primers were biotinylated at the5′ end 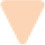; Probes 12-IP1 and 23-IP1 were labeled with a FAM hapten at the5′ end 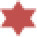, whereas probes 12-DP1 and 23-DP1 were labeled with a Dig hapten at the5′ end 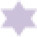. All probes contained a 3′ phosphate group modification 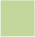 and included an nfo 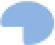 endonuclease recognition site 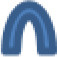; The red and green sequences within the probes sequences indicate bases complementary to the insertion allele.

Based on the bovine *PRNP* gene sequence (GenBank accession No. AJ298878.1) and the sequencing results obtained in this study, DNA samples with distinct genotypes (insertion or deletion) for the 12 bp and 23 bp loci were selected. MIRA primers for each locus were designed using Clone Manager software according to the guidelines provided in the TwistAmp Assay Design Manual. MIRA-LFD probe sequences, ranging from 46 to 52 nucleotides in length, were designed within the MIRA primer amplification region and did not overlap with the specific primer binding sites (48, 49). Each specific probe was modified with a 5′ fluorescein (FAM) or digoxigenin (Dig) label. A tetrahydrofuran (THF) residue, acting as the recognition site for the nfo endonuclease, was incorporated approximately 30 nucleotides from the 5′ end. The THF site was positioned 15 nucleotides upstream from the 3′ end, which was blocked with a C3-spacer. For each locus, an initial set of one forward and three reverse primer combinations was designed and screened for amplification efficiency using standard MIRA. The primer pairs that successfully amplified the target fragment of the expected size, without producing non-specific amplification or primer-dimer artifacts, were selected for subsequent MIRA-LFD assay development (Supplementary Table S1). These optimal pairs were 12-mF1/12-mR2 for the 12 bp locus and 23-mF1/23-mR3 for the 23 bp locus. Corresponding probes for the insertion (12-IP1, 23-IP1) and deletion (12-DP1, 23-DP1) alleles were then designed accordingly. A schematic of the probe design principle is shown in Figure 1, and the final primers and probes used in the assay are listed in Table 2. All oligonucleotides were synthesized by Hangzhou Tsingke Biotechnology Co., Ltd. (China). Primers were diluted to 10 μm and probes to 1 μm, and all were stored at 4 °C until use.

**Table 2:** Sequences of the optimal MIRA primers and probes.

Locus	Primer	Primer sequence (5^′^ → 3^′^)	Fragment length (bp)
12bp	12-mF1	CGGATTGGTGGGAGGCAGACCTTGACCGTGAGTAG	315
	12-mR2	[Biotin]GGCCTCGCCCTTGTTCTTCTGAGCTCCCCA	
	12-IP1	[FAM]TTTACTCGGAATGTGGGCgggggccgcggcHGGCTGGTCCCCCTCC[C3 Spacer]	
	12-DP1	[Dig]GGAGAGCTCCATTTACTCGGAATGTGGGCTHGCTGGTCCCCCTCCC[C3 Spacer]	
23bp	23-mF1	TTTCAAGTCCTCCCAGCCCAGGTGCCAGCCAT	228
	23-mR3	[Biotin]TTTTGTCCTATTCTGGCTATTGTTGCCATG	
	23-IP1	[FAM]TATCACGTCAAtctcagatgtcttcccaacagcaHCCTCAGACGTCATGGG[C3 Spacer]	
	23-DP1	[Dig]AATTCCAACTCCTAGCTATCACGTCAAGHCTCAGACGTCATGGG[C3 Spacer]	

The reverse primers were biotinylated at the 5' end. Probes 12-IP1 and 23-IP1 were labeled with FAM at their 5' ends, whereas probes 12-DP1 and 23-DP1 were labeled with Dig at their 5' ends. All probes contained a 3' phosphate group. The “H” represents a tetrahydrofuran (THF) residue. Lowercase letters within the probe sequences indicate bases complementary to the insertion allele.

### 2.4 MIRA amplification and product detection

#### 2.4.1 MIRA amplification and primer screening

Basic MIRA reactions were performed using the MIRA90 Kit (Basic Type, Amp-Future (Changzhou) Biotech Co., Ltd.) according to the manufacturer's instructions. Each 50 μl reaction mixture contained 29.4 μl of Buffer A, 2.5 μl of Buffer B, 2.0 μl of forward primer (10 μm), 2.0 μl of reverse primer (10 μm), 5.0 μl of template DNA, and 9.1 μl of ddH_2_O. Amplification was performed in a PCR instrument at 37 °C for 30 min. After amplification, proteins were removed by adding an equal volume of phenol:chloroform:isoamyl alcohol (25:24:1) to the reaction products, followed by vortexing and centrifugation at 12,000 rpm for 5 min. The supernatant was collected and analyzed by electrophoresis on a 2% agarose gel. Primer sets were selected based on the presence of a single specific band of the expected size, with no detectable non-specific amplification or primer-dimer formation.

Subsequently, primer-probe combinations for MIRA-LFD were screened using the DNA Isothermal Rapid Amplification Kit (LFD Type, Amp-Future (Changzhou) Biotech Co., Ltd.). For each target locus, multiple candidate primer sets and allele-specific probes were designed; their complete sequences are listed in Supplementary Table S1. The reaction mixture consisted of 29.4 μl of Buffer A, 2.5 μl of Buffer B, 2.0 μl of forward primer (10 μm), 2.0 μl of reverse primer (10 μm), 0.6 μl of probe (10 μm), 5.0 μl of template DNA, and 8.5 μl of ddH_2_O. The reaction was incubated at a constant 37 °C for 30 min. After amplification, 10 μl of the product was diluted in 90 μl of deionized water. Then, 80 μl of the diluted mixture was applied to a dual nucleic acid detection test strip (Sangon Biotech (Shanghai) Co., Ltd.). Results were visually interpreted after 5 min, with the C line serving as the control line and the T line as the test line.

#### 2.4.2 MIRA condition optimization and duplex LFD design

Based on the results from agarose gel electrophoresis of MIRA products and LFD test strips, the selected specific primer-probe sets were further optimized. To improve the accuracy and sensitivity of the MIRA-LFD detection system, several parameters were systematically evaluated, including amplification temperature (30 °C, 33 °C, 36 °C, 39 °C, 42 °C, 45 °C), reaction time (5, 10, 15, 20, 25, 30 min), and probe ratio (insertion probe: 0.5/0.4/0.3/0.2/0.1 μl; deletion probe: 0.1/0.2/0.3/0.4/0.5 μl). The optimal conditions identified were validated through independent reproducibility experiments, each repeated at least three times.

Given that each of the two target loci can present at least two genotypic scenarios (homozygous or heterozygous), genotyping was performed using a duplex LFD strip (Sangon Biotech (Shanghai) Co., Ltd.). In the primer-probe design, the hapten conjugated to the reverse primer serves as the marker for the control (C) line, while the hapten conjugated to the probe serves as the marker for the test (T) line.

### 2.5 Duplex MIRA-LFD reaction system for the 12 bp polymorphic locus

Based on the optimization results, the final duplex MIRA-LFD system for the 12 bp locus was prepared in a 50 μl reaction volume containing: 29.4 μl Buffer A, 2.5 μl Buffer B, 2.0 μl upstream primer 12-mF1 (10 μm), 2.0 μl downstream primer 12-mR2 (10 μm), 0.4 μl insertion probe 12-IP1 (10 μM), 0.2 μl deletion probe 12-DP1 (10 μm), 5.0 μl template DNA, and 8.5 μl ddH_2_O. The MIRA reaction was performed at 39 °C for 20 min. After amplification, 10 μl of the reaction mixture was diluted in 90 μl deionized water. Then, 80 μl of the diluted solution was applied to the dual nucleic acid detection test strip. Results were interpreted visually by color development after 5 min. A negative control was included by replacing the DNA template with an equal volume of ddH_2_O while keeping all other components and steps identical.

### 2.6 Duplex MIRA-LFD reaction system for the 23 bp polymorphic locus

Based on the optimization results, the final duplex MIRA-LFD system for the 23 bp locus was prepared in a 50 μl reaction volume containing: 29.4 μl Buffer A, 2.5 μl Buffer B, 2.0 μl upstream primer 23-mF1 (10 μm), 2.0 μl downstream primer 23-mR3 (10 μm), 0.3 μl insertion probe 23-IP1 (10 μm), 0.3 μl deletion probe 23-DP1 (10 μm), 5.0 μl template DNA, and 8.5 μl ddH_2_O. The thermal conditions and detection procedure were identical to those described in Section 3.5.

### 2.7 Limit of detection (LOD) analysis for MIRA-LFD

To determine the limit of detection (LOD) of the duplex MIRA-LFD assay, genomic DNA samples representing each of the three genotypes at both the 12 bp and 23 bp loci were selected, with two individuals per genotype. DNA samples of the same genotype were pooled and subjected to LOD testing using 10-fold serial dilutions under the optimized detection conditions.

### 2.8 Detection and analysis of commercial samples

A total of 62 commercially available samples, including raw milk, milk powder, beef, and cheese, were genotyped for *PRNP* polymorphisms using the optimized duplex MIRA-LFD systems for the 12 bp and 23 bp loci. In parallel, three samples per genotype were randomly selected for sequencing verification as described in Section 3.2.

Based on the duplex MIRA-LFD results, allele and genotype frequencies were calculated for the 12 bp and 23 bp indel polymorphisms.

## 3 Results

### 3.1 DNA extraction results

The concentration and purity of the 62 extracted DNA samples were determined. For meat samples, the DNA concentration was 46.2 ± 9.55 ng/μl with an OD_260_/_280_ ratio of 1.93 ± 0.07. Raw milk samples had a DNA concentration of 12.7 ± 1.09 ng/μl and an OD_260_/_280_ ratio of 1.66 ± 0.21. For milk powder samples, the DNA concentration was 6.9 ± 2.65 ng/μL with an OD_260_/_280_ ratio of 1.74 ± 0.23. PCR amplification confirmed that all extracted DNA samples produced specific amplification of the *PRNP* gene fragment, indicating that the concentration and purity of the DNA extracted from each sample type were suitable for subsequent PCR analysis.

### 3.2 Sequencing analysis of the two polymorphic loci in the PRNP gene

**Figure 2:**
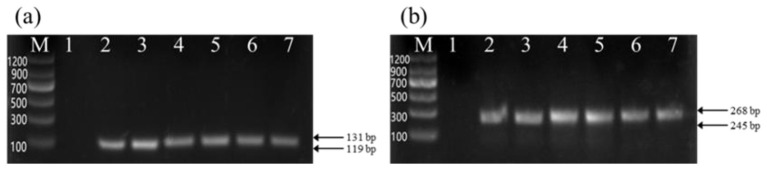
PCR products of the 12 bp and 23 bp polymorphic loci of the PRNP gene. **(A)** PCR products amplified with the 12indel FR primers; **(B)** PCR products amplified with the 23indel FR primers. Lane M represents the DNA Marker C (100–1,200 bp). Lane 1: negative control. Lanes 2–3: genotype of (–/–); Lanes 4–5: genotype of (+/–); Lanes 6–7: genotype of (+/+).

**Figure 3:**
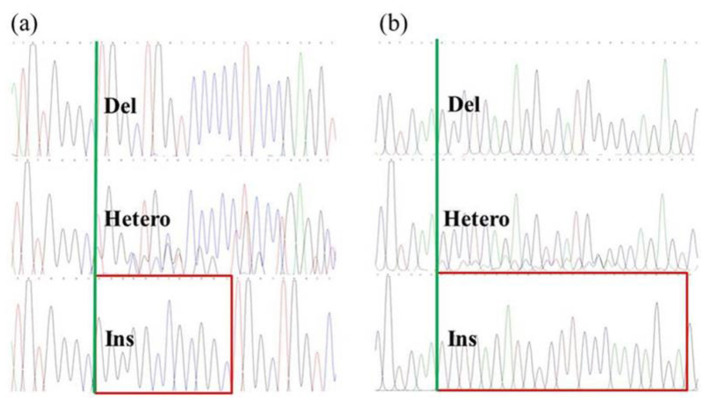
Sequencing results of the 12 bp and 23 bp polymorphic loci in the PRNP gene **(A)** Sequencing chromatograms for the 12 bp indel polymorphism. **(B)** Sequencing chromatograms for the 23 bp indel polymorphism. Del represents (–/–); Hetero represents (+/–); Ins represents (+/+).

Electrophoretic analysis of the PCR products for the *PRNP* gene showed fragment sizes of approximately 119/131 bp for the 12 bp locus and 245/268 bp for the 23 bp locus (Figure 2), which matched the expected sizes. However, due to the small size differences, different genotypes could not be reliably distinguished by electrophoresis alone. Sequencing confirmed three genotypes for both the 12 bp and 23 bp polymorphisms: homozygous deletion (–/–), homozygous insertion (+/+), and heterozygous indel (+/–) (Figure 3).

### 3.3 Screening results of MIRA primers and probes

**Figure 4:**
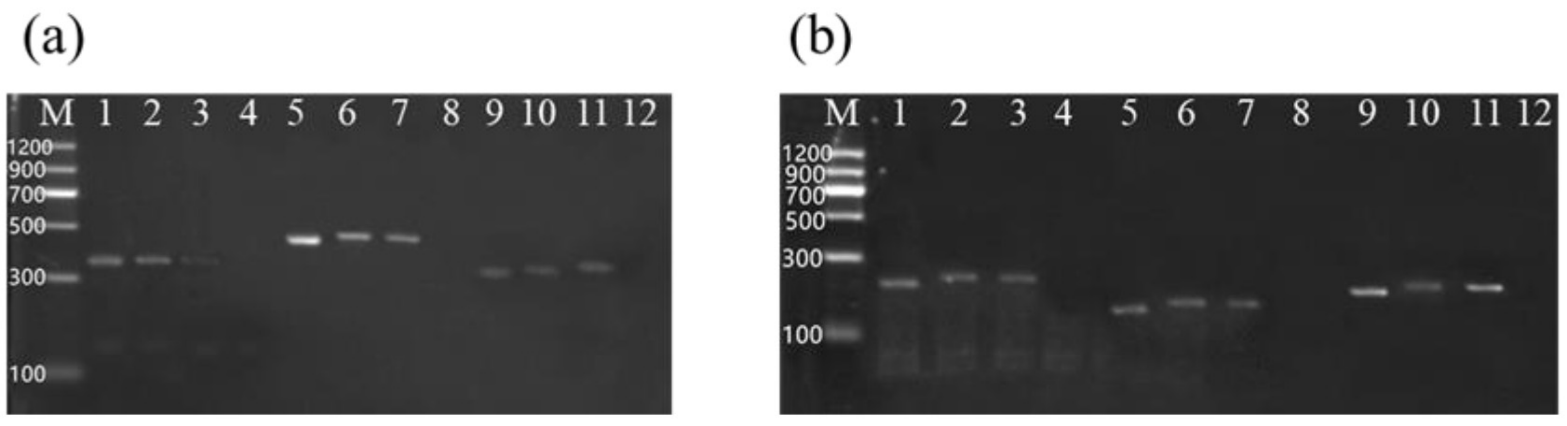
MIRA primers screening results of the 12 bp and 23 bp loci of the PRNP gene. Lane M: DNA Marker C (100–1,200 bp). **(A)** Lanes 1–4: primer set 12-mF1/12-mR1; Lanes 5–8: primer set 12-mF1/12-mR2; Lanes 9–12: primer set 12-mF1/12-mR3. **(B)** Lanes 1–4: primer set 23-mF1/23-mR1; Lanes 5–8: primer set 23-mF1/23-mR2; Lanes 9–12: primer set 23-mF1/23-mR3. Lanes 1, 5, 9: (–/–) samples; Lanes 2, 6, 10: (+/–) samples; Lanes 3, 7, 11: (+/+) samples; Lanes 4, 8, 12: negative controls.

**Figure 5:**
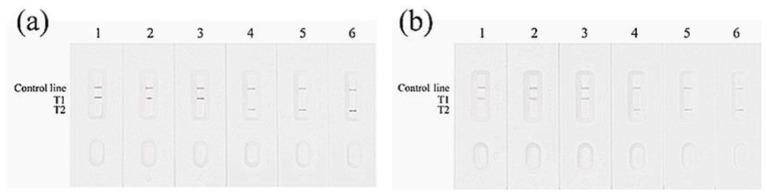
MIRA-LFD probes screening results of the 12 bp and 23 bp loci of the PRNP gene. **(A)** Lanes 1–3: results with probe 12-IP1; Lanes 4–6: results with probe 12-DP1. **(B)** Lanes 1–3: results with probe 23-IP1; Lanes 4–6: results with probe 23-DP1.

Three designed MIRA primer combinations (12-mF1/12-mR1/12-mR2/12-mR3 and 23-mF1/23-mR1/23-mR2/23-mR3) were tested for amplification of bovine DNA samples (Figure 4). The results indicated that primer sets 12-mF1/12-mR2 (for the 12 bp locus) and 23-mF1/23-mR3 (for the 23 bp locus) produced clear target bands of the expected sizes with no non-specific amplification, performing better than the other primer combinations. The sequences of all candidate primers are listed in Supplementary Table S1. Based on this screening, the reverse primers 12-mR2 and 23-mR3 were 5′-biotinylated. Allele-specific probes were used, with their 5′ ends labeled with FAM (12-IP1, 23-IP1) or Digoxigenin (12-DP1, 23-DP1) and their 3′ ends phosphorylated to prevent extension. The detection results for these specific probes with MIRA products of different genotypes are shown in Figure 5. Probes corresponding to each genotype produced clear visible signals on the lateral flow dipsticks for DNA samples of that genotype. These results demonstrate that the selected primers and probes are suitable for detecting the 12 bp and 23 bp polymorphisms in the bovine *PRNP* gene. Therefore, primer sets 12-mF1/12-mR2 and 23-mF1/23-mR3 were chosen as the optimal MIRA primers for subsequent assays.

### 3.4 Duplex MIRA-LFD detection system for the 12 bp and 23 bp polymorphic loci

**Figure 6:**
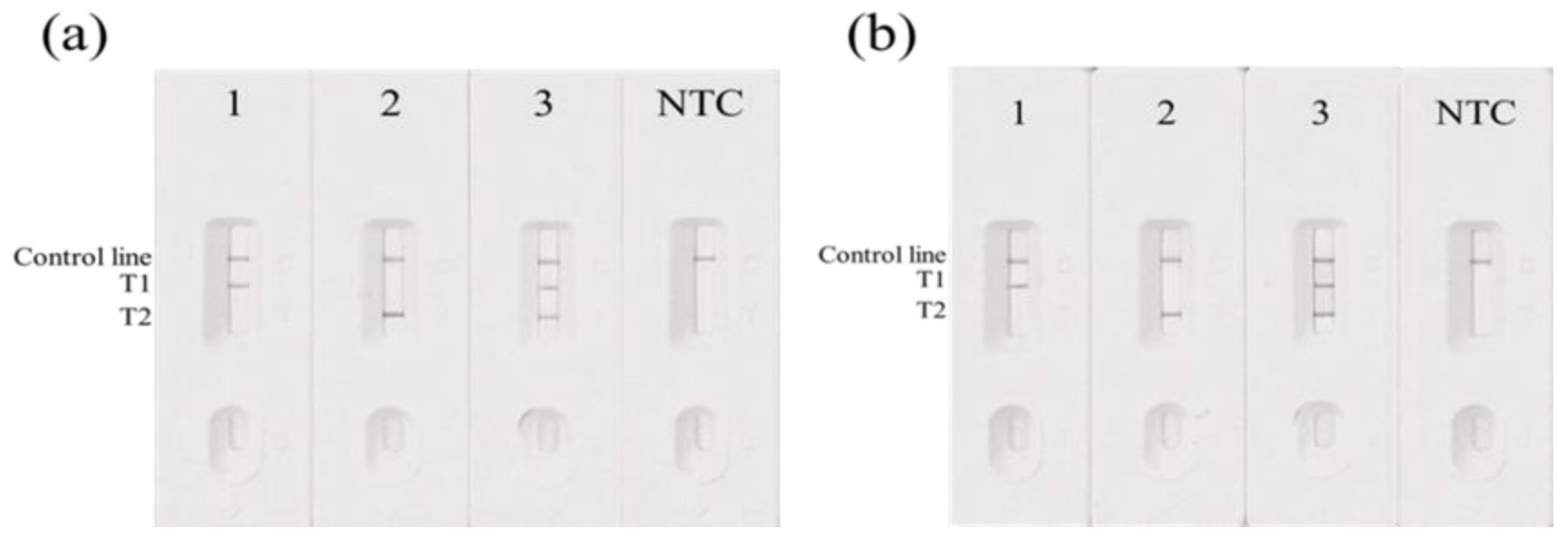
Duplex MIRA-LFD genotyping results for the 12 bp **(A)** and 23 bp **(B)** polymorphic loci. Strip 1: (+/+) genotype; Strip 2: (-/-) genotype; Strip 3: (+/-) genotype; NTC: no-template control.

Based on DNA sequencing results and the basic MIRA-LFD reaction system, DNA templates representing the three genotypes at both the 12 bp and 23 bp polymorphic loci were selected to develop the duplex MIRA-LFD system. Results showed that all tested samples produced a positive control signal on the LFD strips for both polymorphisms. For the homozygous deletion genotype, the positive amplification product produced only two visible lines: the control line (C) and the test line T2. For the homozygous insertion genotype, only the control line (C) and test line T1 appeared. Indel heterozygous samples displayed three visible lines: the control line (C), test line T1, and test line T2. A negative result showed only the control line (C). The LFD results are presented in Figure 6. These results clearly distinguished the different genotypes at the two polymorphic loci, indicating that the established duplex MIRA-LFD detection method is convenient, efficient, and highly accurate.

### 3.5 Limit of detection of MIRA-LFD

**Figure 7:**
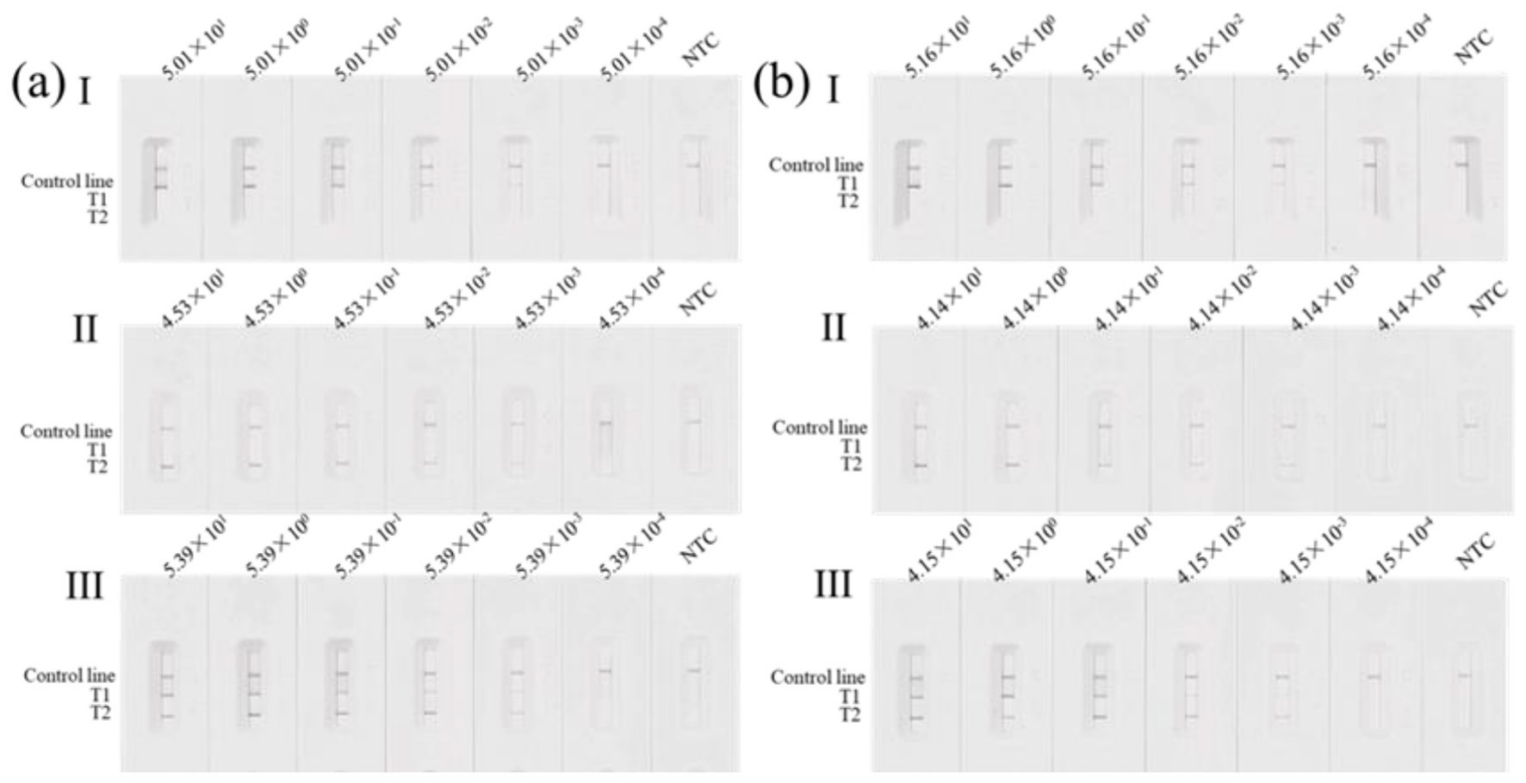
LOD analysis of the duplex MIRA-LFD for the 12 bp **(A)** and 23 bp **(B)** polymorphic loci. **(I)** (+/+) genotype; (II) (-/-) genotype; (III) (+/-) genotype; NTC: no-template control.

The limit of detection (LOD) of the duplex MIRA-LFD assay was determined using serial dilutions of DNA templates representing the three different genotypes at the 12 bp and 23 bp polymorphic loci, as previously identified by sequencing. Results are shown in Figure 7. Signal intensity on the test lines gradually decreased with declining DNA concentration.

The calculated LODs based on the amount of DNA added were as follows. For the 12 bp locus, the LOD was 5.01 × 10^−3^ ng/μl (final concentration in the reaction system: 5.01 × 10^−4^ ng/μl) for the homozygous insertion genotype, 4.53 × 10^−3^ ng/μl (4.53 × 10^−4^ ng/μl in the reaction system) for the homozygous deletion genotype, and 5.39 × 10^−3^ ng/μl (5.39 × 10^−4^ ng/μl in the reaction system) for the heterozygous genotype.

For the 23 bp locus, the LOD was 5.16 × 10^−3^ ng/μl (5.16 × 10^−4^ ng/μl in the reaction system) for the homozygous insertion genotype, 4.14 × 10^−3^ ng/μl (4.14 × 10^−4^ ng/μl in the reaction system) for the homozygous deletion genotype, and 4.15 × 10^−3^ ng/μl (4.15 × 10^−4^ ng/μl in the reaction system) for the heterozygous genotype.

These results show that the lowest detectable concentrations for all genotypes were well below the typical concentrations of the extracted DNA, confirming that the DNA extracted using the commercial kit is fully compatible with the MIRA-LFD system established in this study.

### 3.6 Detection of commercial samples

**Figure 8:**
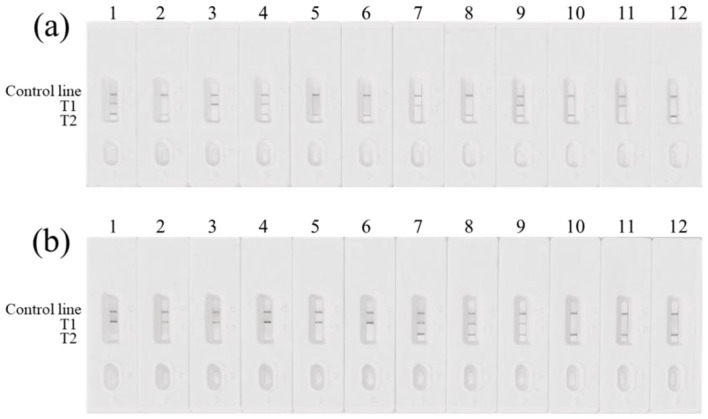
MIRA-LFD results for partial commercial samples showing the 12 bp and 23 bp indel polymorphisms. **(A)** Strips 3, 7, 11 represent 12 bp (+/+) genotypes; Strips 2, 5, 6, 8, 9, 10, 12 represent 12 bp (-/-) genotypes; Strips 1, 4, 9 represent 12 bp (+/-) genotypes. **(B)** Strips 1–6 represent 23 bp (+/+) genotypes; Strips 10–12 represent 23 bp (-/-) genotypes; Strips 7–9 represent 23 bp (+/-) genotypes.

The 62 commercially available cattle-derived products were analyzed using the developed duplex MIRA–LFD assay. For the 12 bp locus, 18 samples were identified as homozygous deletion, 13 as homozygous insertion, and 31 as heterozygous. For the 23 bp locus, 12 samples were homozygous deletion, 9 were homozygous insertion, and 41 were heterozygous. Partial genotyping results are shown in Figure 8. The LFD detection results were completely consistent with the DNA sequencing results, confirming that the method provides an accurate and visual approach for genotyping the bovine *PRNP* gene.

Based on the MIRA–LFD results, allele and genotype frequencies were calculated for the 12 bp and 23 bp indel polymorphisms. In the tested commercial samples, the genotype frequency for the 12 bp locus was 21.9% for homozygous insertion, 28.9% for homozygous deletion, and 49.2% for heterozygous indel. For the 23 bp locus, the genotype frequency was 20.9% for homozygous insertion, 27.9% for homozygous deletion, and 51.2% for heterozygous indel. Differences in polymorphism and genotype frequencies between the two loci are presented in Table 3.

**Table 3:** Allele and genotype frequencies of insertion/deletion polymorphisms in the bovine PRNP gene.

Locus	*n*	Allele frequency		Genotype frequency		
		Insertion Allele	Deletion Allele	+/+	+/-	-/-
12 bp+/-	62	0.460	0.540	0.219	0.492	0.289
23 bp+/-		0.465	0.535	0.209	0.512	0.279

## 4 Discussion

Since the full-length sequencing of the bovine *PRNP* gene was completed, substantial advances have been achieved in understanding its genetic diversity (22). To date, more than 60 polymorphic sites have been reported. Among these, the 23 bp indel in the promoter region and the 12 bp indel are key genetic regulators of BSE susceptibility. Epidemiological and genetic association analyses indicate that individuals carrying either the homozygous deletion or heterozygous genotype at the 23 bp locus are at higher risk of developing the disease (3). Similarly, individuals with deletions at the 12 bp locus also show increased susceptibility. Conversely, individuals carrying insertion alleles at both the 12 bp and 23 bp loci exhibit the strongest resistant phenotype, whereas those homozygous for the deletion at both loci show the highest susceptibility (50). The distribution of these polymorphisms differs globally among cattle breeds. The relatively high frequency of the D12D23 haplotype (double deletion) in European cattle herds correlates with the high incidence of BSE in these populations. In contrast, Southeast Asian cattle breeds and buffalo populations (e.g., the nearly 100% frequency of the I12I23 haplotype in Guizhou buffalo) demonstrate inherent advantages in disease resistance (51–53).

Molecular mechanism studies indicate that the 12 bp deletion disrupts the binding site for the SP1 transcription factor in the *PRNP* gene, resulting in transcriptional repression of the prion protein. The 23 bp deletion interferes with the binding of the RP58 repressor protein to the *PRNP* gene, inducing chromatin conformational reprogramming. Together, these two mechanisms synergistically regulate the *PRNP* expression network and cellular prion protein (PrP^C^) metabolic homeostasis, ultimately influencing the accumulation and transmission efficiency of the pathogenic prion protein (PrP^Sc^) (7, 20, 54, 55). Therefore, indel polymorphisms in the bovine *PRNP* gene are highly relevant for disease-resistant breeding strategies and serve as valuable auxiliary markers for selecting resistant individuals.

Current genotyping methods for these two indel loci primarily include molecular techniques such as Sanger sequencing (21, 29–34), PCR-RFLP analysis (35), and TaqMan probe-based qPCR (36), as well as next-generation sequencing (NGS) (37) and microarray genotyping (38). However, these approaches are generally limited by long processing times, high cost, low throughput, and the requirement for specialized personnel, which restricts their application in routine livestock farm settings and basic laboratory environments.

The MIRA-LFD method established in this study expands the range of available detection technologies. It offers simplicity, speed, and reliability, thereby improving the efficiency and potential for wider application of bovine *PRNP* genotyping. As an advancement in isothermal amplification, MIRA enables efficient amplification of target sequences at ambient temperatures (43, 56). In terms of assay design, MIRA technology is compatible with multiple detection formats, including agarose gel electrophoresis, fluorescent probes, and lateral flow dipsticks. Through cooperative design of specific molecular probes targeting the THF site, which is specifically cleaved by the nfo endonuclease (57), combined with an LFD-based colorimetric readout (58), result visualization and improved detection sensitivity were achieved. Analyzing its technical features, the MIRA-LFD system provides advantages such as broad temperature adaptability, low cost, and visual interpretation. The technology has now been commercialized and applied in various fields. For example, in clinical medicine, Hu et al. (59) established a MIRA-LFD system that detects *Acinetobacter baumannii* in blood specimens within 15 min. Sun et al. (43) applied MIRA-LFD for hepatitis B virus detection, achieving 100% detection accuracy. In food safety monitoring, Yi et al. (48) developed a method for *Bacillus cereus* with a limit of detection (LOD) of 12 fg/μl DNA. In species identification, Sun et al. (60) established a field-ready assay targeting the *CytB* gene, greatly facilitating on-site testing. In genotyping, Li et al. (61) used this approach to identify codon mutations linked to drug-resistance genes, and Zhang et al. (49) developed a triple MIRA-LFD assay for *Y-chromosome* genotyping. In summary, the development and technical strengths of MIRA-LFD make it a highly suitable platform for point-of-care testing applications.

Based on the MIRA-LFD technology platform, this study established a genotyping method for the 12 bp and 23 bp indel polymorphisms in the bovine *PRNP* gene, enabling efficient detection in cattle and cattle-derived products. The method is simple, low–cost, and highly efficient. The detection process and results showed that the duplex MIRA-LFD reaction system, operating at a constant 39 °C, completes DNA amplification and detection within 30 min. It achieved a minimum detection limit of 4.14 × 10^−3^ ng/μl for sample DNA and exhibited high accuracy in discriminating 12 bp and 23 bp indel genotypes. Compared with conventional techniques, the approach developed here enables rapid and intuitive genotyping of both polymorphic loci, offering an efficient solution for on-site and high-throughput screening applications.

We also observed that nucleic acid test strips are highly sensitive and can be prone to interference from amplification dimers, which may lead to false-positive signals. However, this can be mitigated by optimizing probe concentrations to reduce non-specific reactions. Additionally, considering the potential competition between the two allele-specific probes at the same locus in the duplex MIRA-LFD system, we optimized the concentration ratio of the insertion and deletion probes. The results showed that for the 12 bp locus, the optimal addition volumes were 0.4 μl for the insertion probe and 0.2 μl for the deletion probe (ratio 2:1). For the 23 bp locus, both probes performed best at 0.3 μl each (ratio 1:1), providing optimal overall detection performance. Regarding the limit of detection for genomic DNA, the LOD of 4.14 × 10^−3^ ng/μl obtained in this study is comparable to the LOD reported by Wang et al. (62) for MIRA-LFD detection of *Anisakis simplex*. This detection limit is well below the DNA concentrations extracted from all samples tested, confirming that the method is sufficiently sensitive for practical sample analysis.

Although the current study employed a conventional DNA extraction method such as a centrifugal column kit requiring approximately 40 min, further simplification of the sample preparation step would enhance the field applicability of the duplex MIRA-LFD assay. Several rapid and non-invasive DNA extraction strategies have been reported. These could be integrated with our method. For instance, alkaline lysis methods can be completed within 10 min at room temperature and have been successfully applied to bovine meat samples (63, 64). In addition, hair follicles represent a non-invasive sample source that has been validated for DNA extraction in cattle (65). Notably, a duplex RPA-LFD assay with a 1-min DNA extraction procedure has been developed for meat species identification, achieving a total turnaround time of less than 20 min (66). These findings collectively suggest that optimizing the DNA extraction workflow would enable our MIRA-LFD system to achieve on-farm rapid testing, particularly when using non-invasive samples such as hair follicles or buccal swabs combined with alkaline methods. This provides a promising direction for future research.

**Figure 9:**
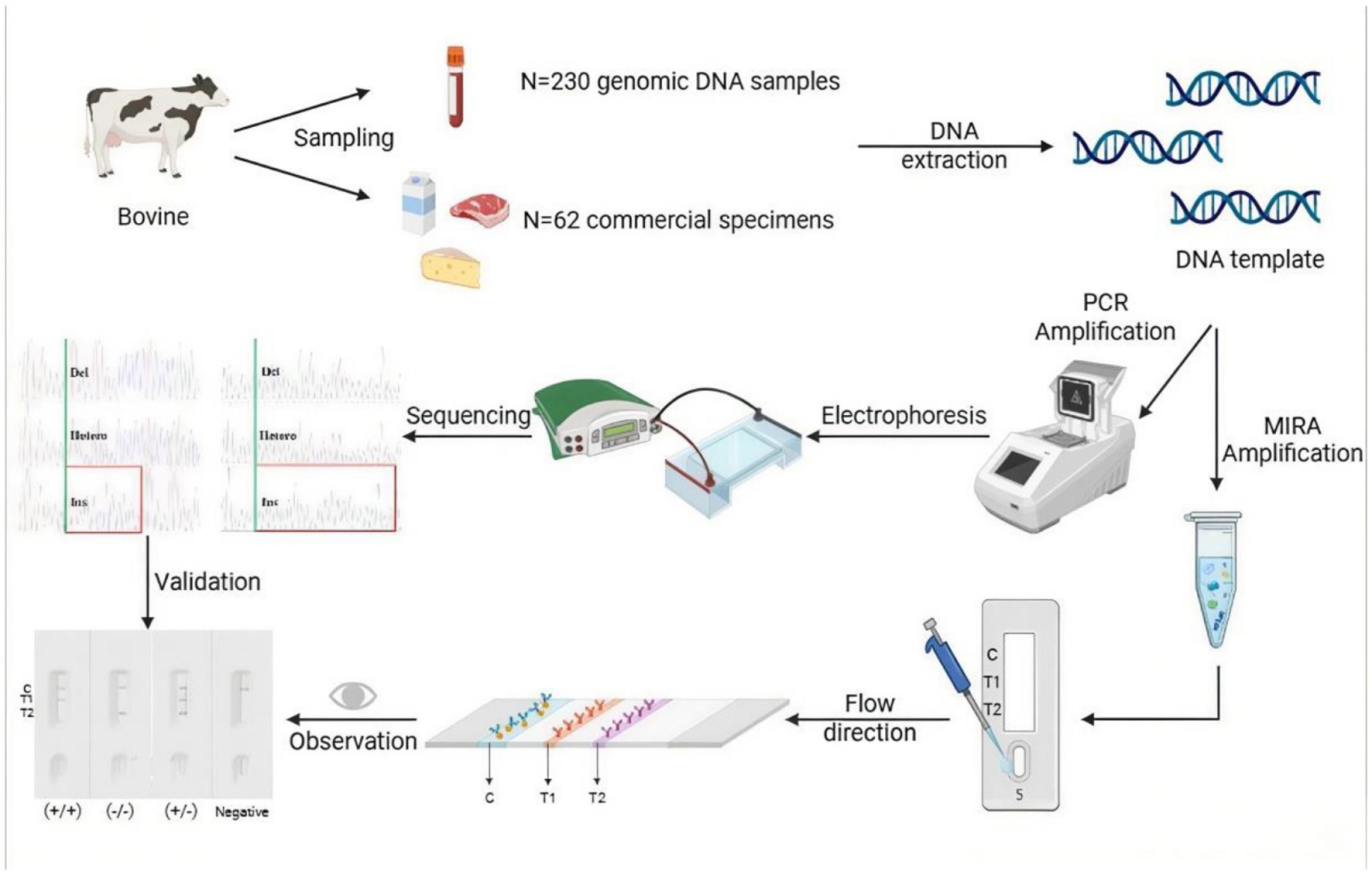
Scheme summarizing the entire process.

In summary, this study targeted the 12 bp and 23 bp indel sequences in the bovine *PRNP* gene, designed specific primers and probes, and established a duplex MIRA-LFD detection method based on MIRA technology (Figure 9). The optimal reaction conditions were systematically screened and optimized. The method offers broad temperature adaptability, high specificity, and visual result interpretation. Thus, the MIRA-LFD approach developed here improves detection efficiency, expands the range of available detection techniques, and enables potential field-ready rapid testing. It provides a practical strategy for rapid screening of disease-resistant cattle and holds considerable scientific and societal relevance.

## 5 Conclusion

In this study, a duplex MIRA-LFD system was constructed and optimized to establish an efficient and convenient genotyping method for the 12 bp and 23 bp indel polymorphisms in the bovine *PRNP* gene. The method is low-cost, highly efficient, and provides visual readout, thereby expanding the range of available detection technologies. This approach has important implications for gene-assisted breeding in cattle and for the safety evaluation of cattle-derived food products.

## Data Availability

The raw sequence data reported in this paper have been deposited in the Genome Sequence Archive (Genomics, Proteomics & Bioinformatics 2025) in National Genomics Data Center (Nucleic Acids Res 2025), China National Center for Bioinformation/Beijing Institute of Genomics, Chinese Academy of Sciences (GSA: accession number CRA030580) that are publicly accessible at https://ngdc.cncb.ac.cn/gsa. All data generated or analysed in this study are available.

## Supplementary material

Supplementary Table S1 Candidate primer and probe sequences screened for the MIRA-LFD assay.
