# Supplementary material for: A rapid visual detection method for indel polymorphisms in the bovine PRNP gene based on a duplex MIRA-LFD assay
Source: Front Vet Sci. 2026 May 1;13:1798131. doi: 10.3389/fvets.2026.1798131 (PMC13176257; doi:10.3389/fvets.2026.1798131)
Supplement: Supplementary Table S1 — Candidate primer and probe sequences screened for the MIRA-LFD assay. [file Table_1.docx]

Supplementary Material

# Supplementary Tables

Supplementary Table S1. Candidate primer and probe sequences screened for the MIRA-LFD assay.

| **Locus** | **Primer** | **Primer Sequence (5’→3’)** | **Fragment length (bp)** |
| --- | --- | --- | --- |
| 12 bp | 12-mF1 | CGGATTGGTGGGAGGCAGACCTTGACCGTGAGTAG | 306 |
|  | 12-mR1 | CTTGTTCTTCTGAGCTCCCCAGCGGTTCCT |  |
|  | 12-mF1 | CGGATTGGTGGGAGGCAGACCTTGACCGTGAGTAG | 315 |
|  | 12-mR2 | [Biotin]GGCCTCGCCCTTGTTCTTCTGAGCTCCCCA |  |
|  | 12-mF1 | CGGATTGGTGGGAGGCAGACCTTGACCGTGAGTAG | 274 |
|  | 12-mR3 | GACCTGCGGCTCCTCTACCGGTGCGATTCG |  |
|  | 12-IP1 | [FAM]TTTACTCGGAATGTGGGCgggggccgcggcHGGCTGGTCCCCCTCC[C3 Spacer] |  |
|  | 12-DP1 | [Dig]GGAGAGCTCCATTTACTCGGAATGTGGGCTHGCTGGTCCCCCTCCC[C3 Spacer] |  |
| 23 bp | 23-mF1 | TTTCAAGTCCTCCCAGCCCAGGTGCCAGCCAT | 234 |
|  | 23-mR1 | ATTACCTTTTGTCCTATTCTGGCTATTGTT |  |
|  | 23-mF1 | TTTCAAGTCCTCCCAGCCCAGGTGCCAGCCAT | 209 |
|  | 23-mR2 | TTGTTGCCATGTAGCAACTACTCCAAAACT |  |
|  | 23-mF1 | TTTCAAGTCCTCCCAGCCCAGGTGCCAGCCAT | 228 |
|  | 23-mR3 | [Biotin]TTTTGTCCTATTCTGGCTATTGTTGCCATG |  |
|  | 23-IP1 | [FAM]TATCACGTCAAtctcagatgtcttcccaacagcaHCCTCAGACGTCATGGG[C3 Spacer] |  |
|  | 23-DP1 | [Dig]AATTCCAACTCCTAGCTATCACGTCAAGHCTCAGACGTCATGGG[C3 Spacer] |  |

The reverse primers were biotinylated at the 5' end. Probes 12-IP1 and 23-IP1 were labeled with FAM at their 5' ends, whereas probes 12-DP1 and 23-DP1 were labeled with Dig at their 5' ends. All probes were modified with a phosphate group at the 3' end. The "H" represents a tetrahydrofuran (THF) residue. Lowercase letters within the probe sequences denote bases complementary to the insertion sequence.
